# Morbidity in California giant salamander (*Dicamptodon ensatus* Eschscholtz, 1833) caused by *Euryhelmis* sp. Poche, 1926 (Trematoda: Heterophyiidae)

**DOI:** 10.1016/j.ijppaw.2024.100908

**Published:** 2024-02-17

**Authors:** Jaimie L. Miller, Lawrence Erickson, Susanne Fork, Constance L. Roderick, Daniel A. Grear, Rebecca A. Cole

**Affiliations:** a U.S. Geological Survey National Wildlife Health Center, Madison, WI, USA; b Department of Pathobiological Sciences, School of Veterinary Medicine, University of Wisconsin-Madison, Madison, WI, USA; c Elkhorn Slough National Estuarine Research Reserve, Watsonville, CA, USA; d Boulder Creek, CA, USA

**Keywords:** Amphibia, California giant salamander, *Dicamptodon ensatus*, *Euryhelmis* sp., Metacercaria, Trematoda

## Abstract

In the fall of 2021, California Department of Fish and Wildlife reported larval and adult California giant salamanders (*Dicamptodon ensatus* Eschscholtz, 1833) with skin lesions at multiple creeks in Santa Clara and Santa Cruz Counties, California, USA. Field signs in both stages included rough, lumpy textured skin, and larvae with tails that were disproportionately long, flat, wavy, and flaccid. Presence of large-bodied larvae suggested delayed metamorphosis, with some larvae having cloudy eyes and suspected blindness. To determine the cause of the disease, three first-of-the-year salamanders from one location were collected, euthanized with 20% benzocaine, and submitted for necropsy to the U.S. Geological Survey, National Wildlife Health Center. Upon gross examination, all salamanders were emaciated with no internal fat stores, and had multiple pinpoint to 1.5-mm diameter raised nodules in the skin over the body, including the head, gills, dorsum, ventrum, all four limbs, and the tail; one also had nodules in the oral cavity and tongue. Histologically all salamanders had multiple encysted metacercariae in the dermis, subcutis, and skeletal muscles of the head, body, and tail that were often associated with granulomatous and granulocytic inflammation and edema. A small number of encysted metacercariae or empty cysts were present in the gills with minimal inflammation, and rarely in the kidney with no associated inflammation. Morphology of live metacercariae (Trematoda: Heterophyiidae), and sequencing of the 28S rRNA gene identified a species of *Euryhelmis* (Poche, 1926). Artificial digestion of a 1.65 g, decapitated, eviscerated carcass yielded 773 metacercariae, all of similar size and morphology as the live specimens. Based on these findings, the poor body condition of these salamanders was concluded to be due to heavy parasite burden. Environmental factors such as drought, increased temperature, and overcrowded conditions may be exacerbating parasite infections in these populations of salamander.

## Highlights

• Morbidity and mortality event affecting California giant salamanders in California.
• Salamanders had rough, lumpy skin, abnormal tails, and delayed metamorphosis.
• *Euryhelmis* sp. metacercariae infection in dermis, subcutis, and skeletal muscles.
• Environmental factors may exacerbate parasite infection in these salamanders.

## Graphical abstract

**Figure:**
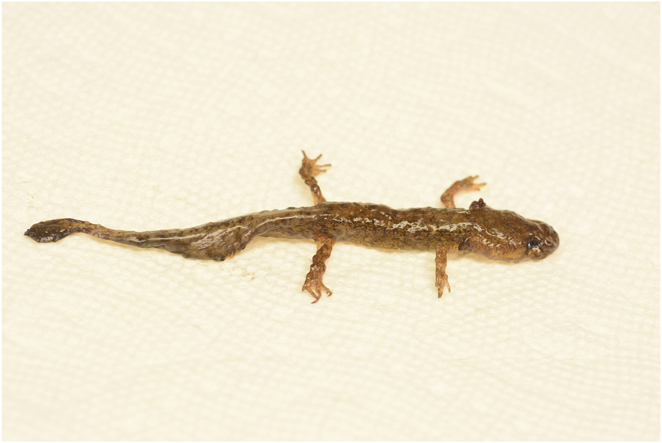

## 1 Introduction

### 1.1 Amphibian decline

Across the globe, amphibian species are in decline (Stuart et al., 2004; Wake and Vredenburg 2008) with up to 42 percent of amphibian species in the United States listed as threatened or declining (Bradford 2005, Grant et al., 2016). Many stressors have been identified as drivers of the declines including habitat loss, climate change, and invasive species which can all play a role in disease risks to amphibians (Blaustein et al., 2011; Hof et al., 2011, Grant et al., 2016). Fungal pathogens such as chytrid (*Batrachochytrium dendrobatidis* Longcore et al., 1999 (Bd) and *B. salamandivorans* Martel et al., 2013 (Bsal)) (Longcore et al., 1999; Fisher et al., 2012; Scheele et al., 2019) and viruses such as ranavirus and *Ambystoma tigrinum* Green, 1825 virus (Jancovich et al., 1997; Earl et al., 2016) have had widespread impacts on amphibian health and may be especially troublesome for species with small population sizes. The role of helminths in amphibian mortalities is not as well studied and tends to be more complicated as the life cycles of helminth parasites are complex and often respond individually and in synergism with the host response to environmental stressors (Tinsley, 1995; Grant et al., 2016; Keller et al., 2021). In addition, disease impacts do not only include mortality but also reduced fecundity, increased predation, depressed growth, or other hard to measure outcomes that can depress a population of free ranging animals.

### 1.2 California giant salamander natural history

**Fig. 1:**
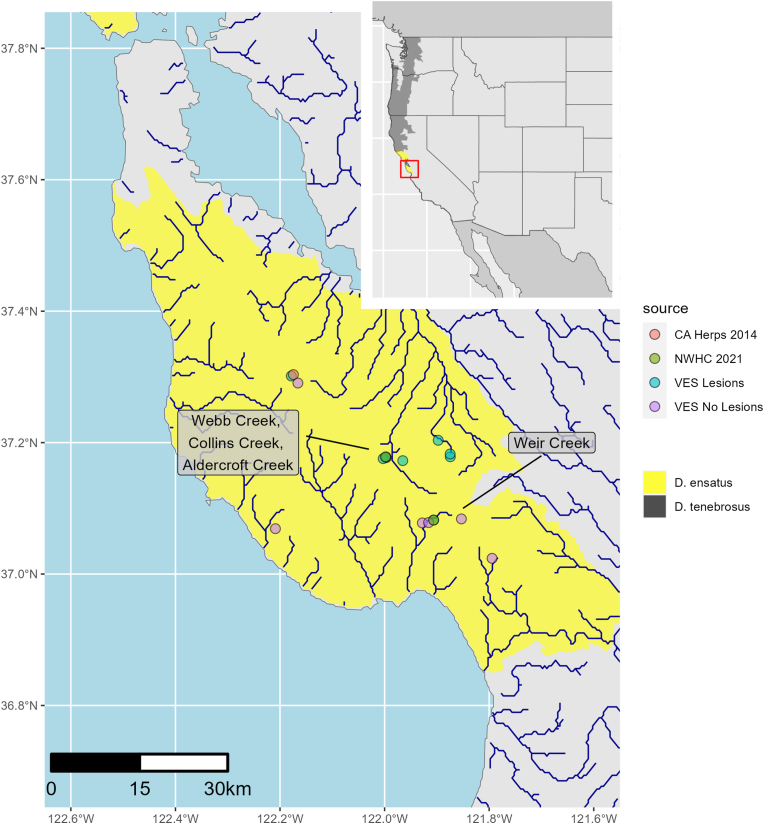
Collection locations of diseased California giant salamanders (*Dicamptodon ensatus*) used for post-mortem investigation (U.S. Geological Survey National Wildlife Health Center), subsequent visual encounter surveys (VES) of *D. ensatus* with (VES Lesions) and without (VES No Lesions) similar clinical skin lesions, and previous visual encounter (CA Herps (https://californiaherps.com/);). *D. tenebrosus* range estimates according to IUCN (2022) and *D. ensatus* range estimates according to the California Department of Fish and Wildlife (Gogol-Prokurat, 2016).

The California giant salamander (*Dicamptodon ensatus* Eschscholtz, 1833) is one of the largest terrestrial salamanders in North America with a range of less than 20,000 square kilometers that encompasses a narrow coastal region of mesic forests from Santa Cruz County north to Mendocino County, California, USA (Fig. 1 inset; Petranka, 2010; Stebbins, 2003; International Union for Conservation of Nature (IUCN), 2022 Gogol-Prokurat, 2016). Overall, little is known about the life history of *D. ensatus,* and much of what is thought to be known has been derived from studies of its close relative to the north, the coastal giant salamander (*D. tenebrosus* Baird and Girard, 1852), which had long been considered as the same species (Nafis, 2023). Adults range between 17 and 30 cm in total length and are one of the few salamanders that can vocalize (Stebbins and Cohen, 1995). Food items include invertebrates and small vertebrates including amphibians, mammals, and reptiles (Antonelli et al., 1972; Bury, 1972). Adults are primarily nocturnal and inhabit damp forests near cold, clear rocky streams or seepages (Stebbins and McGinnis, 2012; Nafis, 2023). Breeding occurs in permanent, small to medium sized rocky streams. Larvae are of the stream-type with short bushy, dull red gills and a tail fin that begins at the base of the hind limbs and extends posteriorly to the tail tip (Petranka, 2010). The post-metamorphosed terrestrial salamanders are far less abundant than the aquatic larvae (Petranka, 2010). The aquatic larval stage is approximately 18 months with metamorphosis occurring at 130–140 mm in total length (Petranka, 2010). Paedomorphosis, or the retention of larval characteristics in sexually mature animals, occurs in this species but its prevalence is unknown (Thompson et al., 2016). *D. ensatus* was listed as near threatened by the IUCN in 2004 because of habitat loss (Hammerson and Bury, 2004). In 2012, the United States Fish and Wildlife Service (USFWS) received a petition requesting *D. ensatus* be listed as threatened or endangered under the U.S. Endangered Species Act, but the petition did not provide substantial information warranting listing (Nature Serve, 2023). Beginning in 2013, collection of *D. ensatus* required a sportfishing license and based upon several factors, including limited range and insufficient knowledge of their basic biology, were designated as a California Species of Special Concern (Thompson et al., 2016; Nafis, 2023).

### 1.3 Case history

**Fig. 2:**
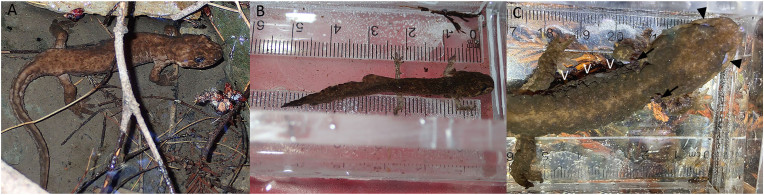
(A) Adult California giant salamander (*Dicamptodon ensatus*) found mid-day in a calm pool. Note the emaciated body condition and granular textured skin. Weir Creek, Santa Cruz County, California, USA. Photo credit: L. Erickson. (B) First year larval stage *D. ensatus*. Note the thin wavy tail with its length longer than the snout to vent length (SVL). Weir Creek, Santa Cruz County, California, USA. Photo credit: L. Erickson (Independent contractor). (C) Second-year larval stage *D. ensatus* with gross disfiguration and nodules (arrows) present on the gills. Note the cloudy appearance of the eyes (arrowheads). Aldercroft Creek, Santa Clara County, California, USA. Photo credit: S. Fork, (Elkhorn Slough National Estuarine Research Reserve).

In early November 2021, while conducting site evaluation for larval salamander population density studies, adult, and larval *D. ensatus* in Webb Creek, Collins Creek, and Aldercroft Creek in the Bear Creek Redwoods Preserve (37.178165 N, 121.99839 W) in Santa Clara County and Weir Creek (37.08213 N, 121.90623 W; Fig. 1) in the Soquel Demonstration State Forest in Santa Cruz County, California, USA were observed with skin lesions and atypical behavior. Adults were emaciated with rough, lumpy textured skin and were found exposed mid-day in calm, shallow water and made no attempt at fleeing when detected (Fig. 2). Subsequent visual encounters noted some *D. ensatus* with similar clinical presentation and some without lesions and normal appearance (Fig. 1). Visual encounters of similarly diseased *D. ensatus* were noted in 2014 (Fig. 1). Adults appeared lethargic, emaciated, flaccid, and had lumpy skin (Fig. 2A). Larvae presented with nodular skin lesions and tails that were disproportionately long, flat, wavy, and flaccid (Fig. 2B). Some larvae had disfigured digits and nodules on the gills with some of the larger larvae displaying opaque or white lenses of the eyes which may have indicated visual limitation (Fig. 2C). Both first- and second-year larvae were observed, as well as larvae that were of a size that would have typically metamorphosed, suggesting there was a retardation in development. Individuals of this species are normally secretive and shelter under rocks, but these were found exposed in calm water, typically in pools. Herein we present this case to highlight the importance of current and historical field observations of biologists along with complete postmortem examinations to identify causes of morbidity and mortality in salamanders.

## 2 Methods

### 2.1 Collection of salamanders

Three immature salamanders with gross skin lesions were collected November 1, 2021 and euthanized by field biologists using a topical 20% benzocaine and shipped chilled by overnight carrier to the U.S. Geological Survey, National Wildlife Health Center (NWHC) Madison, Wisconsin, USA, for necropsy and morbidity investigation. Specimens were collected and euthanized under direction by the California Department of Fish and Wildlife for salvage of moribund animals (L. Erickson Scientific Collection Permit, 192110001-19211-001).

### 2.2 Postmortem examination

Carcass weight, straight line snout to vent length, total length, body condition, and postmortem condition were recorded. General appearance of tissues and organs along with gross lesions or abnormalities were noted as to distribution, severity and, where appropriate, size. Tissue samples from the skin, gills, liver, gall bladder, kidney, spleen, heart, gastrointestinal tract, pancreas, head, and a limb from each carcass were preserved in 10% neutral buffered formalin, processed routinely, embedded in paraffin, sectioned at 5 μm, and stained with hematoxylin and eosin (H&E) for histopathological examination (Luna, 1968).

### 2.3 Microparasite/pathogen analysis

Skin swabs were collected from the dorsum and ventrum to test for the presence of Bd and Bsal by quantitative real-time polymerase chain reaction (qrt-PCR) as described in Blooi et al. (2013, 2016). A pooled sample of liver, kidney, and spleen from one animal (001) and a pooled sample of liver and kidney from the other two animals (002, 003) was tested for the presence of *Ranavirus* sp. (Family Iridoviridae) by qrt-PCR (Leung et al., 2017).

### 2.4 Morphological and molecular characterization of metacercariae

Using a stereo microscope (Leica M165 C, Danahaer Corp Deerfield, Illinois, USA) metacercariae were dissected from the three chilled carcasses. Many of the metacercariae were still alive and manually excysted using a 22-gauge needle, examined using Normarski differential contrast microscopy (Olympus BX50F microscope, Olympus, Center Valley, Pennsylvania, USA). A portion of excysted metacercariae were fixed in 70% ethanol with a coverslip in place, dehydrated is a series of ethanols, stained with Semichon's acid carmine stain (Sigma, Millipore Sigma, Burlington, Massachusetts, USA) cleared in cedarwood oil (Millipore Sigma, Burlington, Massachusetts, USA) then temporarily mounted in cedarwood oil and photographed (Olympus DP74 Color CMOS with CellSens STD software Olympus, Waltham, Massachusetts, USA). To estimate the total number of metacercariae, a decapitated, eviscerated, thawed carcass weighing 1.65 g (animal 001) was digested in a pepsin hydrochloric acid solution (Ash and Orihel, 1987) for 4 hours to free encysted metacercariae from host tissue, and a subset of metacercariae were visually examined as described above.

A subsample of live metacercariae were lysed and DNA extracted using Qiagen DNeasy Blood and Tissue Kit (Qiagen Inc., Valencia, California, USA) per manufacturer instructions. Methods for amplification and sequencing of partial 28S rRNA gene were followed as in Tkach et al. (2003, 2016) with the following modifications: 1x GoTaq® Green Master Mix, M712 (Promega, Madison, Wisconsin, USA) with cycling conditions of 3 min denaturation hold at 94 °C; 40 cycles of 30 s at 94 °C, 30 s at 53 °C and 2 min at 68 °C with final extension of 5 min at 68 °C. The partial 18S rRNA gene was sequenced using two PCR reactions. The first reaction used primers NSF4/18 and NSR 1438/20R (Sato et al., 2010; Tamaru et al., 2015) for amplification with the following modifications: 1x of 10X ExTaq Buffer; 0.2mM dNTP mixture; 0.058U/μl TaKaRa ExTaq® DNA polymerase (Takara Bio, San Jose, California, USA) with cycling conditions of 3 min denaturation hold at 94 °C; 40 cycles of 45 s at 94 °C, 1 min at 50 °C and 90 s at 72 °C with final extension of 7 min at 72 °C. Sequencing used the amplification primers in addition to primers NSF573/19 and NSR 581/18 (Sato et al., 2010). The second PCR reaction used primers NSF573/19 (Sato et al., 2010) and SSU 18R (Tamaru et al., 2015) with the same modifications as noted above for amplification. Amplification of partial cytochrome oxidase c subunit I (COI) utilized Dice1F and Dice11R primers for both amplification and sequencing as in the supplemental chart in Van Steenkiste et al. (2015) with the modification of using 1x of GoTaq® Green Master Mix, M712 (Promega, Madison, Wisconsin, USA). All PCR products were analyzed on a 1% agarose gel, with excess primers and nucleotides removed from the products using ExoSap-IT following manufacturer's instructions (Applied Biosciences Affymetric Inc., Santa Clara, California, USA), followed by DNA sequencing (Functional Biosciences, Madison, Wisconsin, USA) using ABI 3730 xl DNA sequencer automated DNA sequencing instrument (Applied Biosystems, Foster City, California, USA). Resulting sequences were aligned, manually trimmed, and pairwise comparisons made using Seq Man Pro (Lasergene17, DNASTAR, Inc, Madison, Wisconsin, USA). Comparison of sequences in this study were made to sequences via the BLAST method in GenBank (https://www.ncbi.nlm.nih.gov/genbank/). Sequences from GenBank with 98–100% coverage and 94% or higher identity to this study's sequences were chosen for use in phylogenetic comparison, aligned using CLUSTAL (Kumar et al., 2018) then manually trimmed. A phylogenetic tree was constructed using maximum likelihood phylogenetic relationships among each of the gene sequences under the Hasegawa-Kishino-Yano model with 1000 bootstrap replications using MEGA X (Kumar et al., 2018). Cluster analyses were performed by using the unweighted pair group method with arithmetic mean (UPGMA) and neighbor joining algorithms. Statistical support for groupings was estimated by bootstrap analysis (Kumar et al., 2018).

All whole biological (parasite and host) and DNA vouchers were deposited with the Museum of Southwestern Biology, University of New Mexico, Albuquerque, USA (MSB 46087-99; 46100-19). Partial gene sequences obtained from this study were deposited in GenBank (18S rRNA: PP086009, PP086010, PP086017; 28S rRNA: PP086515, PP086516, PP086519, PP086521, PP104327, PP105461, PP105527, PP104404; COI: PP086647, PP086657, PP108742). Supporting data files are available (Cole, 2024, https://doi.org/10.5066/P13DTR5V).

## 3 Results

### 3.1 Postmortem examination and histological findings

**Fig. 3:**
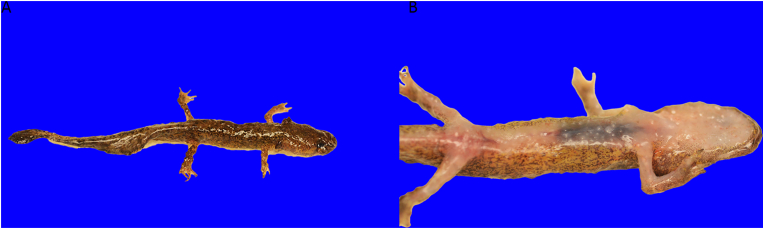
Photographs of the dorsum (A) and ventrum (B) of a euthanized, chilled larval California giant salamander (*Dicamptodon ensatus*) from a morbidity event in Santa Clara County, California, USA. There are numerous pinpoint to 1.5-mm diameter nodules in the skin over the body including the head, gills, dorsum, ventrum, all four limbs, and tail causing a granular texture to the body. Photo credit: J. L. Miller (USGS).

The three euthanized and chilled salamanders were in fair to poor post-mortem condition and emaciated with no internal fat bodies (Table 1). All carcasses had numerous pinpoint to 1.5-mm diameter nodules in the skin over the body including the head, gills, dorsum, ventrum, all four limbs, and tail (Fig. 3A–B). One salamander (001) had similar nodules in the oral cavity and tongue. Both corneas in all the carcasses were clear. All carcasses had food content in the stomach and distal large intestine while two carcasses (002 and 003) had content in the small intestine. One carcass (003) had 12–24 insect larvae in the digestive tract. The size of internal organs was within normal limits and unremarkable.

**Table 1:** Morphometrics, body condition, and postmortem condition of three euthanized, chilled larval California giant salamanders (*Dicamptodon ensatus*) from a morbidity event in Santa Clara and Santa Cruz Counties, California, USA, submitted for necropsy.

Carcass	001	002	003
Weight (g)	3.35	1.59	1.14
Snout-Vent Length (mm)	48	32	32
Total Length (mm)	99	67	53
Sex	U	U	U
Body Condition	E	E	E
Postmortem Condition	F	P	P

U Undetermined Juvenile, E Emaciated, F Fair, P Poor.

**Fig. 4:**
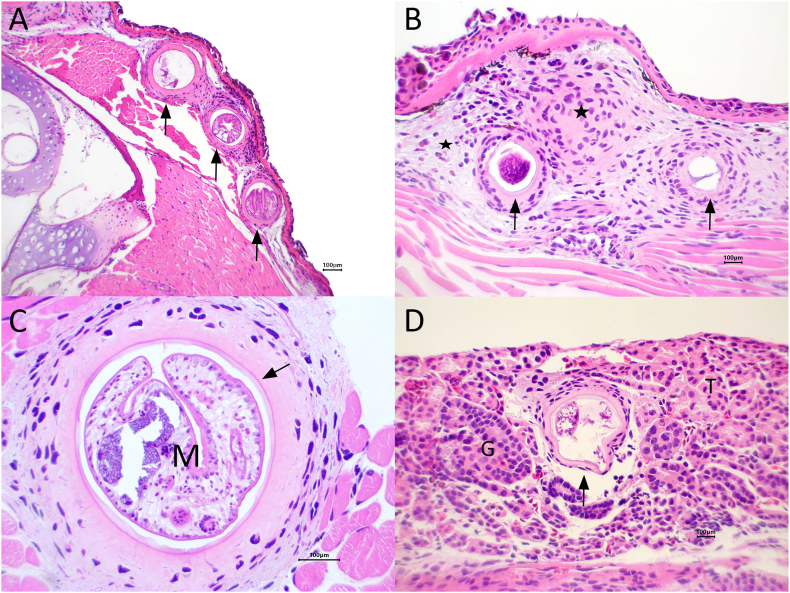
Photomicrographs from euthanized, chilled larval California giant salamanders (*Dicamptodon ensatus*) from a morbidity event in Santa Clara and Santa Cruz Counties, California, USA. (A) Encysted metacercariae (arrows) in the subcutis of the head are surrounded by inflammation and cause undulation of the skin. Cartilage and bone of the skull are to the left. (B) Skin with two encysted metacercariae (arrows) in the subcutis surrounded by macrophages, heterophils, edema (stars). Note the elevation of the epidermis. (C) Skeletal muscle with a metacercaria (M) within a cyst wall (arrow) surrounded by a few macrophages. (D) Kidney with an encysted metacercaria (arrow) in the interstitium that is surrounded by a few macrophages. A glomerulus (G) and tubules (T) are unaffected. Photo credit: J. L. Miller (USGS).

Microscopically, all salamanders had multiple encysted metacercariae or empty cysts in the dermis, subcutis, and skeletal muscles of the head, body, and tail that were often associated with granulomatous and granulocytic inflammation and edema (Fig. 4A–C). A small number of similar encysted metacercariae or empty cysts were present in the gills of all salamanders and, rarely, in the kidney of one salamander, with minimal granulomatous associated inflammation (Fig. 4D). Livers from all three carcasses had moderate, multifocal intrahepatocellular protozoal parasites 2–4 μm in diameter with no inflammation or tissue reaction.

### 3.2 Microparasite/pathogen detection

Neither Bsal nor Bd were detected on swabs taken from the three carcasses. *Ranavirus* sp. was not detected in any of the pooled tissue samples.

### 3.3 Macroparasite characterization

**Fig. 5:**
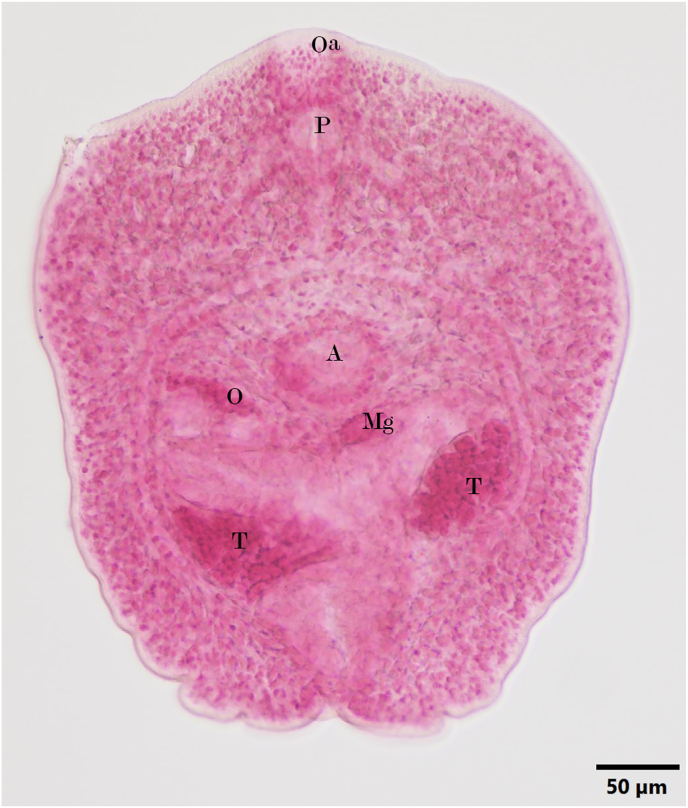
*Euryhelmis* sp. excysted, metacercaria collected from tail skin of a euthanized, chilled larval California giant salamander (*Dicamptodon ensatus*). A = acetabulum; Mg = Mehlis' gland; O = ovary; Oa = oral acetabulum; P = pharynx; T = testis. Photo credit: R. A. Cole (USGS).

Live excysted metacercariae from both muscle and skin changed shapes as they moved. Metacercariae were broader than long, pentagonal in shape with a Y-shaped excretory bladder filled with black granular material, intestinal ceca with lens shaped material, two lobate testes, one club shaped ovary, and ventral acetabulum located at midbody similar in size to the oral acetabulum. Live metacercariae and stained specimens (Fig. 5) were most similar to metacercariae of *Euryhelmis squamula* Rudolphi, 1819 as described in Grabda-Kazubska (1980). A total of 773 metacercariae were counted from the eviscerated, digested carcass with examination of a subset of metacercariae, which had similar size and morphology as those examined and subjected to DNA sequencing. Neither kidney tissue nor liver tissue was available for examination; therefore, no morphological nor genetic characterization of metacercariae or protozoa was possible.

**Fig. 6:**
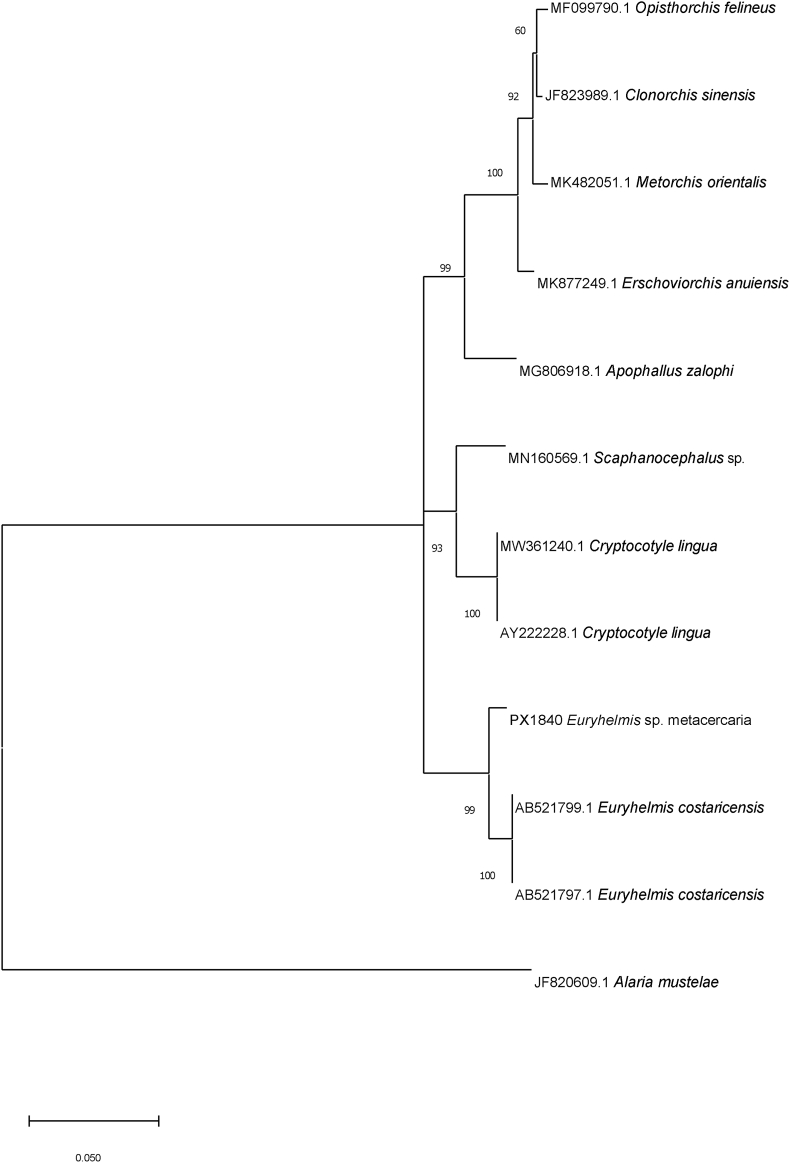
Molecular phylogenetic analysis by Hasegawa-Kishino-Yano method with 1000 bootstrap replications based on partial 28S rRNA gene sequence from a metacercaria identified as *Euryhelmis* sp. removed from the subcutaneous skin of a dead, chilled California giant salamander (*Dicamptodon ensatus*) from a morbidity event in Santa Clara and Santa Cruz Counties, California, USA, and sequences available in GenBank. *Alaria mustelae* is the outgroup. The analysis involved 12 nucleotide sequences with a total of 1125 positions in the final dataset.

Pairwise sequence alignment (Seq Man Pro software DNA Star Lasergene17, DNASTAR, Madison, Wisconsin, USA) of the 10 partial 28S rRNA gene sequences from both skin/subcutis or skeletal muscle were all identical with zero base substitutions (Kumar et al., 2018). GenBlast® (https://www.ncbi.nlm.nih.gov/genbank/) of the partial 28S rRNA, 1220 base pair sequence from metacercaria (PX 1840) removed from the body wall muscle had a 98.44% identity (1201/1220 bp with 1 gap) with an adult stage of *E. costaricensis* Brenes et al., 1960 (GenBank AB521800) and was used in the construction of the phylogenetic tree (Fig. 6) that showed our specimens formed a sister group to the *E. costaricensis* (GenBank ^AB521799.1^.and AB521797.1) Partial (1717 bp) 18S rRNA gene sequence (PX 1823) had a 99% identity (1529/1541 bp with 1 gap) with the same accession. The partial COI sequence (PX 1828) most closely matched *Amphimerus* sp. Barker, 1911 (GenBank MK238506) with 447/534 bp, 84% identity as there were no COI sequences from species of *Euryhelmis* publicly available.

Discussion:

Species of *Euryhelmis* follow a typical digenean life cycle involving aquatic snails as first intermediate hosts; anurans, urodelans, and fish as second intermediate hosts; and mammals as definitive hosts (Kim et al., 2021). Eight species of *Euryhelmis* are currently recognized (Kim et al., 2021) based on the morphology of adult specimens. Four of the eight species also have descriptions of the metacercarial stage. Metacercariae of *E*. *monorchis* Ameel (1938) have one testis and can infect green frog (*Lithobates clamitans*) tadpoles in experimental infections (Ameel, 1938). Metacercarial stages of *E. zelleri* Grabda-Kazubska (1980) possess two round/oval testes, a parallelogram-like body shape with the anterior and posterior margins of the body parallel (Grabda-Kazubska, 1980). *Euryhelmis cotti* Simon (1972) metacercariae have two ovoid testes, a club shaped ovary, a ventral acetabulum 50% smaller than the oral acetabulum, and encyst in muscles of sculpins (*Cottus* sp.; Simon, 1972). However, no shape description was provided nor were voucher specimens deposited. Metacercariae of *E*. *pacificus* Senger and Macy (1952) is pyriform in shape with two ovoid or lobed testes and club-shaped ovary (Senger and Macy, 1952). Adult *E. pyriformis* Webster and Wolfgang (1956) have only one testis and therefore are most likely similar to *E. monorchis* in the metacercarial stage. Morphology of the metacercariae we found infecting *D. ensatus* from California was most similar to the description of *E. squamula* in Grabda-Kazubska (1980) with pentagonal body shape (Fig. 5B), two lobed testes, and oral and ventral acetabulum of similar size with the latter at the midline. The 28S rRNA sequences did not match (1201/1220 bp) with sequences from adult *E. costaricensis*. With no infection trials to produce adult parasite specimens, nor publicly available gene sequences from *E. squamula*, identification to species is not possible.

The life cycles of *E. squamula* on the Pacific coast in the United States requires the freshwater snail *Pristinicola hemphilli*, Pilsbry, 1890 as first intermediate host and amphibian second intermediate hosts such as the coastal tailed frogs (*Ascaphus truei* Stejneger, 1899), red-legged frogs (*Rana aurora* Baird and Girard, 1852), and Cascades frogs (*R*. *cascadae* Slater, 1939; Senger and Macy, 1952; Anderson and Pratt, 1965). In Virginia, USA, *E. squamula* has been reported from northern leopard frogs (*Lithobates pipiens* Schreber, 1782) (McIntosh, 1936). Regardless of the specific identity of the *Euryhelmis*, infection with similar species is likely ubiquitous in amphibians in this area and throughout North America. The original description of *E. pacificus* from *D. tenebrosus* by Senger and Macy (1952; given the coastal Oregon collection site this salamander was most likely not *D. ensatus* as reported) collected in streams from the coast and Cascade Mountain range in Oregon, USA, reported pyriform shaped metacercariae in striated muscle only, noting the site of infection and second intermediate host species as life cycle characteristics specific to *E. pacificus*. In addition, Senger and Macy (1952) noted that 90% of the 50 salamanders they examined were infected and those larger than 50 mm long were always infected. Senger and Macy (1952) noted the average infection was approximately 50 metacercariae (ranging from five to several hundred) and that other amphibians collected in the same areas concurrently were not infected with *E. pacificus*. Schell (1964) reported that the larval and adult stages of the coastal tailed frogs (*A. truei*) from Washington, USA, and Idaho, USA, had metacercariae (not described) in the dermis, subcutaneous connective tissue, muscles, and serous membranes that, when fed to domestic kittens (*Felis catus*), produced both adult *E. pacificus* and *E. squamula* in the intestines 17- and 30-days post infection. Species of *Euryhelmis* have also been described globally, including *E. squamula* in Europe infecting anurans (Grabda-Kazubska, 1980); *E. costaricensis* infecting Morrocan painted frogs (*Discoglossus scovazzi* Camerana, 1878) and North African fire salamanders (*Salamandra algira* Bedriaga, 1883) in Morocco (Díaz-Rodríguez et al., 2018) and *E. costaricensis* metacercarial infections causing disease and mortalities in Tōhoku salamanders (*Hynobius lichenatus* Boulenger, 1883) in Japan (Sato et al., 2010; Ihara, 2010).

Histological study characterized the granulomatous and granulocytic inflammatory and edema in response to the metacercarial cysts throughout the dermis, subcutaneous tissue, and skeletal musculature, occasionally in the gills, and rarely in the kidney. This infection and tissue reaction are the cause of the nodular appearance of the skin and gills seen in the field. Between 30 and 68% of oxygen exchange and 31–86% of carbon dioxide exchange occurs via the skin in salamanders (Duellman and Trueb, 1994; Jørgensen, 2000). Oxygen uptake through the skin is passive and dependent on the proximity of capillaries and blood flow (Duellman and Trueb, 1994); therefore, injury or compromise to capillary beds due to metacercariae or resulting inflammation would reduce gas exchange. Likewise, the space occupying mass of metacercariae in the gills and minor inflammation would lessen efficacy of gas exchange of gill tissue. The cause of the cloudy eyes observed in the field remains unknown. Grossly, the corneas of the submitted salamanders were all clear, and no microscopic changes were noted in the eyes.

The salamanders in these river systems have not been closely monitored by biologists, and it is not known whether such severe infections have been increasing or if they are a regular occurrence. Photographs found on the website California Herps (https://californiaherps.com/) that predate our observations show two clinically similar *D. ensatus* reported from Santa Cruz County, California, USA (Fig. 1). We contacted the original source of the images (Ian Gaston) and were told the images were taken around October 2014 in San Mateo County, California, USA at either Lambert or Peters Creek in the Skyline Ridge Open Space Preserve (37.303505 N, 122.174417 W). Subsequent evaluations, conducted by the authors (Fork, S. and Erickson, L.), of other streams in the region presented additional detection of clinically similar *D. ensatus* in Santa Cruz, Santa Clara, and San Mateo Counties in California, USA. These additional site evaluations were conducted by teams of two to three people involving visual encounter and rock flipping and replacement techniques searching for *D. ensatus*, and when found, visually evaluating all specimens for signs of infection. Clinically similar salamanders have been detected in streams from four different drainages, with the distance between the two farthest locations being approximately 34 km (Fig. 1). All sites evaluated were within contiguous United States (CONUS) Climate Division California (4) Central Coast Drainage (National Oceanic and Atmospheric Administration, 2023). At all sites where *D. ensatus* infected with metacercariae have been found, nearly all individuals observed were infected as indicated by nodular lesions contrasted with the overall low number of salamander observations in streams with a healthy population.

Driving factors that may cause increased occurrence of metacercarial infections, especially at scales that span multiple creeks is not well understood. Crowding of larval amphibians or increased first intermediate (snail) or definitive hosts (e.g., mink, skunks, raccoons, felines) found in this area of California could lead to an increase in infections. Ihara (2010) and Sato et al. (2010) documented increased prevalence and incidence of *E. costariennsis* metacercariae in Tōhoku salamanders (*Hynobius lichenatus*) in the Abukuma Mountains in northeastern Honshu, Japan, over a 10-year timeframe noting that increasing populations of introduced raccoons (*Procyon lotor* L, 1758) and feral American mink (*Mustela vison* Schreber, 1777) from the United States may have relevance to the increased incidence. Environmental conditions such as drought occurring regionally, where infected predators are drawn to limited water sources, could facilitate transmission of large numbers of trematodes. This region had been experiencing prolonged drought conditions for more than a decade prior to these observations. Identifying the definitive hosts of this species of *Euryhelmis* in this system along with prevalence of the snail host would be the first step to begin understanding the infection dynamics and their potential impact on the populations of *D. ensatus*.

## Disclaimer

Any use of trade, firm, or product names is for descriptive purposes only and does not imply endorsement by the U.S. Government.

## References

[bib1] Ameel D. (1938). The morphology and life cycle of *Euryhelmis monorchis* n. sp. (Trematoda) from the mink. J. Parasitol..

[bib2] Anderson G., Pratt I. (1965). Cercaria and first intermediate host of *Euryhelmis squamula*. J. Parasitol..

[bib3] Antonelli A., Nussbaum R., Smith S. (1972). Comparative food habits of four species of stream-dwelling vertebrates (*Dicamptodon ensatus, D. copei, Cottus tenuis* and *Salmo gairdneri*). Northwest Sci..

[bib4] Ash L., Orihel T. (1987).

[bib5] Blaustein A., Han B., Relyea R., Johnson P., Buck J., Gervasi S., Kats S. (2011). The complexity of amphibian population declines: understanding the role of cofactors in driving amphibian losses. Ann. N. Y. Acad. Sci..

[bib6] Blooi M., Pasmans F., Longcore J., Spitzen-van Der Sluijs A., Vercammen F., Martel A. (2013). Duplex Real-Time PCR for rapid simultaneous detection of *Batrachochytrium dendrobatidis* and *Batrachochytrium salmandrivorans* in amphibian samples. J. Clin. Microbiol..

[bib7] Blooi M., Pasmans F., Longcore J., Spitzen-van Der Sluijs A., Vercammen F., Martel A. (2016). Correction for Blooi et al., Duplex Real-Time PCR for rapid simultaneous detection of *Batrachochytrium dendrobatidis* and *Batrachochytrium salmandrivorans* in amphibian samples. J. Clin. Microbiol..

[bib8] Bradford D., Lannoo M. (2005). Status and Conservation of United States Amphibians.

[bib9] Bury B. (1972). Small mammals and other prey in the diet of the Pacific giant salamander (*Dicamptodon ensatus*). Am. Midl. Nat..

[bib11] Cole R. (2024). The Bear Creek Redwoods Preserve in Santa Clara County, California.

[bib12] Díaz-Rodríguez J., Donaire-Barroso D., Jowers M. (2018). First report of *Euryhelmis* parasites (Trematoda, Heterophyidae) in Africa: conservation implications for endemic amphibians. Parasitol. Res..

[bib13] Duellman W., Trueb L. (1994).

[bib14] Earl J., Chaney J., Sutton W., Lillard C., Kouba A. (2016). Ranavirus could facilitate local extinction of rare amphibian species. Oecologia.

[bib15] Fisher M., Henk D., Briggs C., Brownstein J., Madoff L., McCraw S., Gurr S. (2012). Emerging fungal threats to animal, plant and ecosystem health. Nature.

[bib16] Gogol-Prokurat M. (2016). "California giant salamander range - CWHR A004 [ds1133]." California department of fish and Wildlife. Biogeographic information and observation system (BIOS). http://bios.dfg.ca.gov.

[bib17] Grabda-Kazubska B. (1980). *Euryhelmis zelleri* sp. n. and *Euryhelmis squamula* (Rudolphi, 1819) trematoda, heterophyidae) metacercariae from *Rana temporaria* from the babia gόra national park, Poland. Acta Parasitol. Pol..

[bib18] Grant E., Miller D., Schmidt B., Adams M., Amburgey S., Chambert T., Cruickshank S., Fisher R., Green D., Hossack B., Johnson P., Joseph M., Rittenhouse T., Ryan M., Waddle J., Walls S., Bailey L., Fellers G., Gorman T., Ray A., Pilliod D., Price D., Saenz D., Sadinski W., Muths E. (2016). Quantitative evidence for the effects of multiple drivers on contintental-scale amphibian declines. Sci. Rep..

[bib19] Hammerson G., Bury B. (2004).

[bib20] Hof C., Araújo M., Jetz W., Rahbek C. (2011). Additive threats from pathogens, climate and land-use change for global amphibian diversity. Nature.

[bib21] Ihara S. (2010). Incidence of cutaneous metacercarial nodules in Tōhoku salamanders (*Hynobius lichentatus*) and their recent increase in the Northern region of the Abukuma Mountains. Bull. Herpetol. Soc. Jpn..

[bib22] IUCN (2022). https://www.iucnredlist.org.

[bib23] Jancovich J., Davidson E., Morado J., Jacobs B., Collins J. (1997). Isolation of a lethal virus from endangered tiger salamander *Ambystoma tigrinum stebbinsi*. Dis. Aquat. Org..

[bib24] Jørgensen C. (2000). Amphibian respiration and olfaction and their relationships: from Robert Townson (1794) to the present. Biol. Rev..

[bib25] Keller S., Roderick C., Caris C., Grear D., Cole R. (2021). Acute mortality in California tiger salamander (*Ambystoma californiense*) and Santa Cruz long-toed salamander (*Ambystoma macrodactylum croceum*) caused by *Ribeiroia ondatrae* (Class: trematoda). Int. J. Parasitol.: Paras. and Wildl..

[bib26] Kim H.C., Hong E.J., Ryu S.Y., Park J., Cho J.G., Yu D.H., Chae J.S., Choi K.S., Park B.D. (2021). *Euryhelmis squamula* (Digenea: heterophyidae) recovered from Korean raccoon dog, *Nyctereutes procyonoides koreensis*, in Korea. Kor. J. Parasitol..

[bib27] Kumar, Stecher G., Li M., Knyaz C., Tamura K. (2018). Mega X: molecular evolutionary genetics analysis across computing platforms. Mol. Biol. Evol..

[bib28] Leung W., Thomas-Walters L., Garner T., Balloux F., Durrant C., Price S. (2017). A quantitative-PCR based method to estimate ranavirus viral load following normalisation by reference to an ultraconserved vertebrate target. J. Virol Methods.

[bib29] Longcore J., Pessier A., Nichols D. (1999). *Batrachochytrium dendrobatidis* gen. et sp. nov., a chytrid pathogenic to amphibians. Mycologia.

[bib30] Luna L.G., Blakiston Division (1968). Manual of Histologic Staining Methods of the Armed Forces Institute of Pathology.

[bib31] Martel A., Sptizen-van der Sluijs A., Blooi M., Bert W., Ducatelle R., Fisher M., Woeltjes A., Bosman W., Chiers K., Bossuyt F., Pasmans F. (2013). *Batrachochytrium salmandrivorans* sp. nov. causes lethal chytridiomycosis in amphibians. Proc. Natl. Acad. Sci. USA.

[bib32] McIntosh A. (1936). The occurrence of *Euryhelmis squamula* in the United States. J. Parasitol..

[bib33] Nafis G. (2023). California giant salamander -. Dicamptodon Ensatus.

[bib34] National Oceanic and Atmospheric Administration (2023). https://www.ncei.noaa.gov/access/monitoring/reference-maps/conus-climate-divisions.

[bib35] Petranka J.W. (2010).

[bib36] Sato H., Ihara S., Inaba O., Une Y. (2010). Identification of *Euryhelmis costararicensi*s metacercariae in the skin of Tōhoku hynobiid salamanders (*Hynobius lichenatus*), Northeastern Honshu, Japan. J. Wildl. Dis..

[bib37] Scheele B., Pasmans F., Skerratt L., Berger L., Martel A. (2019). Amphibian fungal panzootic causes catastrophic and ongoing loss of biodiversity. Science.

[bib38] Schell S.C. (1964). *Bunoderella metteri* gen. and sp. n (Trematoda: allocreadiidae) and other trematode parasites of *Ascaphus truei* Stejneger. J. Parasitol..

[bib39] Senger C.M., Macy R.W. (1952). Helminths of Northwest Mammals. Part III., the description of *Euryhelmis pacificus* n. sp. and notes on its life cycle. J. Parasitol..

[bib40] Serve Nature (2023). https://explorer.natureserve.org/Taxon/ELEMENT_GLOBAL.2.105355/Dicamptodon_ensatus.

[bib41] Simon M. (1972).

[bib42] Stebbins R.C. (2003).

[bib43] Stebbins R., Cohen N. (1995).

[bib44] Stebbins R.C., McGinnis S.M. (2012).

[bib45] Stuart S., Chanson J., Cox N., Young B., Roderigues A., Fischman D., Waller R. (2004). Status and trends of amphibians declines and extinctions worldwide. Science.

[bib46] Tamaru M., Yamaki S., Jimenez L.A., Sato H. (2015). Morphological and molecular genetic characterization of three *Capillaria* spp. (*Capillaria anatis, Capillaria pudendotecta*, and *Capillaria madseni*) and *Baruscapillaria obsignata* (Nematoda: trichuridae: Capillariinae in avians. Parasitol. Res..

[bib47] Thompson R.C., Wright A.N., Shaffer H.B. (2016).

[bib48] Tinsley R. (1995).

[bib49] Tkach V., Littlewood D.T.J., Olson P.D., Kinsella J.M., Świderski Z. (2003). Molecular phylogenetic analysis of the microphalloidea ward, 1901. Syst. Parasitol..

[bib50] Tkach V., Kudlai O., Kostadinova A. (2016). Molecular phylogeny and systematics of the echinostomatoidea Looss, 1899 (Platyhelminthes: digenea). Int. J. Parasitol..

[bib51] Van Steenkiste N., Locke S.A., Castelin M., Marcogliese D.J., Abbott C.L. (2015). New primers for DNA barcoding of digeneans and cestodes (Platyhelminthes). Mol. Ecol. Resour..

[bib52] Wake D., Vredenburg T. (2008). Are we in the midst of the sixth mass extinction? A view from the world of amphibians. Proc. Natl. Acad. Sci. U.S.A..

[bib53] Webster G., Wolfgang R.W. (1956). *Alaria canadensis* sp. nov. and *Eurhelmis pyriformis* sp. nov. from the skunk *Mephitis mephitis* in Quebec. Can. J. Zool..

